# Ethnicity and insurance status predict metastatic disease presentation in prostate, breast, and non‐small cell lung cancer

**DOI:** 10.1002/cam4.3109

**Published:** 2020-06-08

**Authors:** Nima Aghdam, Mary McGunigal, Haijun Wang, Michael C. Repka, Mihriye Mete, Stephen Fernandez, Chiranjeev Dash, Waddah B. Al‐Refaie, Keith R. Unger

**Affiliations:** ^1^ Department of Radiation Medicine MedStar‐Georgetown Hospital Washington DC USA; ^2^ MedStar Health Research Institute Hyattsville MD USA; ^3^ NYU Winthrop Hospital New York NY USA; ^4^ Georgetown Lombardi Comprehensive Cancer Center Office of Minority Health & Health Disparities Research Washington DC USA; ^5^ MedStar‐Georgetown Surgical Outcomes Research Center Washington DC USA; ^6^ MedStar‐Georgetown University Hospital Washington DC USA

**Keywords:** breast, insurance and ethnicity interaction, lung, metastatic cancer, prostate

## Abstract

**Background:**

Ethnicity and insurance status have been shown to impact odds of presenting with metastatic cancer, however, the interaction of these two predictors is not well understood. We evaluate the difference in odds of presenting with metastatic disease in minorities compared to white patients despite access to the same insurance across three common cancer types.

**Methods:**

Using the National Cancer Database, a multilevel logistic regression model that estimated the odds of metastatic disease was fit, adjusting for covariates including year of diagnosis, ethnicity, insurance, income, and region. We included adults diagnosed with metastatic prostate, non–small cell lung cancer (NSCLC), and breast cancer from 2004 to 2015.

**Results:**

The study cohort consisted of 1 191 241 prostate cancer (PCa), 1 310 986 breast cancer (BCa), and 1 183 029 NSCLC patients. Private insurance was the most protective factor against metastatic presentation. Odds of presenting with metastatic disease were 0.190 [95% CI, 0.182‐0.198], 0.616 [95% CI, 0.602‐0.630], and 0.270 [95% CI, 0.260‐0.279] for PCa, NSCLC, and BCa compared to uninsured patients, respectively. Private insurance provided the most significant benefit to non‐Hispanic White PCa patients with 81% reduction in odds of metastatic presentation and conferred the least benefit to African‐American NSCLC patients at 30.4% reduction in odds of metastatic presentation.

**Conclusions:**

Insurance status provided the single most protective effect against metastatic presentation. This benefit varied for minorities despite similar insurance. Reducing metastatic disease presentation rates requires addressing social barriers to care independent of insurance.

## Graphical abstract

Ethnicity and insurance status have been shown to impact odds of presenting with metastatic cancer, however the interaction of these two predictors is not well understood. In this national‐level analysis, we found that insurance status provided the single most protective effect against metastatic presentation. This benefit varied for minorities despite similar insurance.

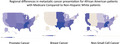

## 1 INTRODUCTION

While overall cancer mortality continues to decline, patients who present with metastatic disease pose a complex clinical challenge.^1^ Across all cancers, metastatic presentation portends worse prognosis and commonly renders a patient incurable. Access to early prevention and diagnostic services is critical in preventing late stage presentation and improving survival outcomes.^2^ Lack of insurance is a significant barrier to both early diagnosis and potential cure.^3^ However, access to care is mediated by factors beyond insurance status, including social determinants of health, namely, ethnicity, income, and geography.^4^, ^5^

Providing health insurance to the previously uninsured aims to bridge the gap in cancer care disparities along socioeconomic strata.^6^ Recent studies reveal increased health services utilization in states with Medicaid expansion.^7^, ^8^ Improved management of chronic illnesses such as diabetes illustrates the benefits of expanded insurance access.^9^ Cancer stage at diagnosis could serve as an indicator for utilization of screening and protend success in curing early‐stage cancers.^10^, ^11^ Recent studies suggest that Affordable Care Act's Medicaid expansion show a greater rates of breast cancer screening and early‐stage diagnosis.^12^

Despite increased access to insurance, delivery of high‐quality care continues to be hampered by entrenched obstacles in minority communities largely due to social determinants of health.^13^ While ethnic minority and lack of insurance have historically predicted for inferior outcomes, there is a paucity of studies examining the interplay between insurance and ethnicity as related to metastatic disease presentation. Furthermore, access to insurance does not always result in utilization of health services, therefore, it is critical to assess the needs of a population not solely based on their insurance status but also social determinants of care. To achieve cancer care equity, it is crucial to fill this knowledge gap by evaluating potential determinants of disparities among diverse patients with a range of insurance access and cancer types. Our hypothesis is that insurance or lack thereof by itself does not lead to favorable cancer presentation. Although we expect this effect to be most pronounced in minority patients who lack insurance, we anticipate that the effect will be present in other forms of insurance holders as well. The goal of our analysis is to identify trends in metastatic disease presentation and examine the role of ethnicity in conjunction with insurance.

## 2 METHOD

### 2.1 Data source

The NCDB is a prospectively collected, hospital‐based registry populated by data generated from the Commission On Cancer (CoC) accredited centers in the US, sponsored by the American College of Surgeons and the American Cancer Society. The database captures approximately 70% of cancers diagnosed in these hospitals. Significant data points related to patient's disease, treatment, and demographic features along with estimates of income and education levels by corresponding zip codes are included.

### 2.2 Ethics statement

Ethical approval was sought and granted from the Institutional Review Board (IRB). After acquiring an IRB exemption, the NCDB for Breast, Prostate and Lung cancers was accessed. The study conforms to Consolidated Standards of Reporting Trials (CONSORT) guidelines. The diagrammatic flowchart is appended in the supplementary material for each disease site (Figure S1).

### 2.3 Study cohort

The NCDB for breast, prostate, and lung cancer was evaluated to identify patient‐related factors that affect metastatic cancer presentation despite access to insurance. Breast and Prostate cancer are unique in that both can be diagnosed early through readily available screening which conceivably would reduce the rate of metastatic presentation.^14^, ^15^ Alternatively, lung cancer typically presents as metastatic. We selected NSCLC (non–small cell lung cancer) as it is the most common subtype. We sought to contrast highly prevalent cancer types with various risk factors, gender specificity, and potential for early detection and cure.

The NCDB for prostate, breast, and lung cancers were queried for all patients ≥ 18 years old from 2004 to 2015. Clinical stage was determined using the 7th Edition of American Join Committee on Cancer (AJCC) staging manual. Patients with noninvasive histology and missing demographic data were excluded. Additional covariates analyzed include age, gender (dichotomized for lung cancer), insurance status, and Charlson Deyo Comorbidity scores. The facility types in the NCDB are as follows: community cancer program, comprehensive community cancer program, academic/research program (includes NCI designated comprehensive cancer centers), integrated network cancer programs, and “other/unknown.”

Outcome variable was the rate of metastatic presentation. This was to capture the most severe instance of cancer care disparity at the onset of an irreversibly poor prognosis. The rate of stage IV presentation is assumed in this analysis to provide a composite endpoint for available determinant of disparity in the database.

### 2.4 Statistical analyses

Comparison of demographic features was made for each site between patients with localized disease (stages I‐III) versus those with metastatic disease (IV). Bivariate analysis was conducted using the Pearson Chi‐Square test to identify differences in sociodemographic features. Multivariable logistic regression was utilized to assess the direct association of insurance with metastatic presentation. Adjusted odds ratio for insurance status, with and without ethnic stratification, was calculated while controlling for facility type and region, median income, comorbidity score, education, demographic area, and year of diagnosis. *P*< 0.001 were considered significant. Finally, subgroup analysis was conducted for each cancer site based on ethnic strata and insurance status with a separate logistic regression model adjusted with the above demographic features within each ethnic cohort. All statistical analyses were performed using SAS 9.4 (Cary, NC). For specific *P*‐values please refer to tables. Patients without available data points for the sociodemographic features used in the analysis were censored per diagram (Figure S1).

## 3 RESULTS

### 3.1 Descriptive analysis

The study cohort consisted of 1 191 241 prostate cancer (PCa), 1 310 986 breast cancer (BCa), and 1 183 029 lung cancer (NSCLC) patients with mean [SD] age of 61.32 [13.64], 68.36 [10.90], and 65.34 [8.98] years, respectively. Overall, 5.7% of PCa, 6% of BCa, and 43.4% of NSCLC patients presented with metastatic disease. Uninsured patients constituted 1.74%, 2.27%, and 3.27% of overall cohort in each site, respectively. Metastatic patients were more likely to be uninsured compared to patients who presented with locoregional disease (Stage I‐III) (for PCa: 5.22% vs 1.53%, for BCa: 6.07% vs 2.04%, and for NSCLC: 4.49% vs 2.33%). (Additional bivariate comparison of patient characteristics is presented in Tables S1.1‐S1.3).

### 3.2 Differences of metastatic presentation in African‐American, Caucasian, and Hispanic patients by cancer site

For patients with PCa and BCa, the rates of metastatic presentation differed significantly between African American (AA), Hispanic (H), and non‐Hispanic white (NHW). 7.63% of AA, 8.93% of H, and 5.31% of NHW patients presented with metastatic PCa. This trend persisted in BCa with 9.68% of AA, 6.69% of H, and 5.77% of NHW presenting with metastatic disease. Rate of metastatic presentation was comparable across ethnic strata in NSCLC with 47% of AA, 49.66% of H, and 42.33% of NHW patients presenting with stage IV disease.

### 3.3 Minority patients presenting with metastatic disease are more frequently uninsured or on Medicaid

There was a significant difference between African‐American, Hispanic, and non‐Hispanic White patients in rate of those who are either uninsured or on Medicaid. This was consistent across disease sites. Overall, 10.8%, 18.58%, and 20.15% of AA with PCa, BCa, and NSCLC patients were uninsured or Medicaid recipients compared to 16.01%, 29.24%, and 22.63% of H patients and 2.39%, 5.87%, and 7.61% of NHW patients in each disease site, respectively. However, Hispanic patients with metastatic presentation were more likely to be uninsured or Medicaid recipients compared to the AA and, in turn, metastatic AA patients were more likely to be uninsured or on Medicaid than NHW with PCa (H: 33.29% vs AA: 23.84% vs NHW: 6.69%) and BCa (H: 42.5% vs AA: 30.63% vs NHW: 13.04%). This trend persisted in NSCLC (H: 27.39% vs AA: 23.57% vs NHW: 9.66%). In breast and prostate cancer, patients who presented with metastatic disease were more likely to reside in zip codes with lower educational attainment and median income. There appeared to be relative parity in these features regardless of stage at presentation for NSCLC patients.

### 3.4 Analyses of effect within each ethnicity identify Insurance as a significant predictor of Metastatic presentation

To determine the role of insurance in odds of metastatic presentation in each cancer site, we analyzed each ethnic group separately stratified by insurance status using a multilevel logistic regression model. Possessing private insurance or Medicaid reduces the odds of presenting with Metastatic PCa in NHW patients by 81.8% and 28.1%, respectively. For AA patients, the reduction is 79.1% and 24.8%, respectively, and for H patients the reduction is 79.1% and 29.9% compared to uninsured patients within the ethnic cohort. For NHW patients with private insurance and Medicaid, the reduction in odds of presenting with Metastatic BCa is 77.3% and 41.6%, respectively. For AA patients the reduction is lower at 70.03% and 37.6%, respectively, and for H patients even lower at 58.7% and 19.5%. Finally, for patients with NSCLC, the risk reduction for NHW patients with private insurance and Medicaid is 39.6% and 26.7% compared to AA patients with 30.4% and 15.1%, respectively, and for H patients the odds are reduced by 42.2% and 25%, respectively. (A Summary table for each ethnicity and disease site is presented in Table 1).

**Table 1:** Logistic regression was performed to determine the extent to which insurance status affects odds of presenting with metastatic Breast Cancer, NSCLC, and Prostate Cancer within each ethnic group

Baseline characteristics	African‐American patients				hispanic patients				non‐hispanic white patients			
	Estimate	95% confidence limits		*P*‐value	Estimate	95% confidence limits		*P*‐value	Estimate	95% confidence limits		*P*‐value
Odds ratio for presentation of metastatic breast cancer												
Facility type												
Community Cancer Program	–	–	–	–	–	–	–	–	–	–	–	–
Academic/Research Program	0.987	0.921	1.058	0.7198	1.182	1.041	1.344	0.0101	0.982	0.952	1.013	0.2598
Comprehensive Community Cancer Program	0.941	0.878	1.01	0.0907	0.899	0.79	1.024	0.1099	0.891	0.865	0.917	<0.0001
Integrated Network Cancer Program	0.992	0.914	1.076	0.8395	1.116	0.958	1.3	0.1594	0.961	0.924	0.999	0.0438
Facility location												
New England	–	–	–	–	–	–	–	–	–	–	–	–
Middle Atlantic	1.365	1.165	1.6	0.0001	1.391	1.073	1.802	0.0127	1.151	1.103	1.201	<0.0001
South Atlantic	1.5	1.27	1.771	<0.0001	1.337	0.803	2.226	0.2637	0.995	0.943	1.05	0.8542
East North Central	1.541	1.315	1.805	<0.0001	1.503	1.185	1.907	0.0008	1.302	1.247	1.359	<0.0001
East South Central	1.587	1.246	2.021	0.0002	1.442	1.113	1.867	0.0056	1.038	0.982	1.098	0.1868
West North Central	1.481	1.243	1.764	<0.0001	1.49	1.179	1.884	0.0009	0.971	0.927	1.016	0.2052
West South Central	1.429	1.223	1.67	<0.0001	1.719	1.349	2.192	<0.0001	1.016	0.973	1.06	0.4851
Mountain	1.43	1.195	1.711	<0.0001	1.036	0.682	1.573	0.8696	1.101	1.046	1.158	0.0002
Pacific	1.506	1.281	1.771	<0.0001	1.62	1.277	2.054	<0.0001	1.005	0.954	1.058	0.8657
Age												
<50	–	–	–	–	–	–	–	–	–	–	––	
50‐59	1.19	1.126	1.258	<0.0001	1.146	1.041	1.262	0.0056	1.31	1.269	1.352	<0.0001
60‐69	1.153	1.085	1.226	<0.0001	1.185	1.065	1.318	0.0018	1.253	1.211	1.295	<0.0001
70+	1.292	1.204	1.386	<0.0001	1.402	1.236	1.591	<0.0001	1.295	1.247	1.345	<0.0001
Charlson comorbidity score												
0	–	–	–	–	–	–	–	–	–	–	–	–
1	0.995	0.947	1.047	0.8537	1.019	0.922	1.126	0.7104	1.106	1.078	1.136	<0.0001
2+	1.306	1.207	1.413	<0.0001	1.372	1.147	1.641	0.0005	1.705	1.635	1.778	<0.0001
Demographic area												
Metropolitan	–	–	–	–	–	–	–	–	–	–	–	–
Rural	0.859	0.695	1.063	0.1623	1.433	0.804	2.554	0.223	0.974	0.907	1.046	0.466
Urban	0.969	0.896	1.049	0.4429	1.227	1.031	1.46	0.0214	0.916	0.89	0.943	<0.0001
Income												
<$38 000	–	–	–	–	–	–	–	–	–	––		
$38 000‐$47 999	0.933	0.885	0.984	0.0103	0.936	0.844	1.037	0.204	0.988	0.956	1.022	0.4864
$48 000‐$62 999	0.896	0.84	0.955	0.0008	0.982	0.879	1.097	0.7461	0.979	0.945	1.014	0.2267
$3 000+	0.849	0.782	0.922	<0.0001	0.897	0.777	1.036	0.1393	0.988	0.95	1.028	0.5497
Percent without high school diploma												
≥21%	–	–	–	–	–	–	–	–	–	–	–	–
13.0%‐20.9%	0.932	0.888	0.979	0.0046	1.031	0.933	1.14	0.5506	0.899	0.869	0.929	<0.0001
7.0%‐12.9%	0.961	0.898	1.028	0.2493	0.951	0.842	1.075	0.4207	0.829	0.801	0.858	<0.0001
<7.0%	0.912	0.827	1.005	0.0634	0.957	0.812	1.128	0.6025	0.71	0.682	0.74	<0.0001
Insurance status												
Uninsured	–	–	–	–	–	–	–	–	–	–	–	–
Private/managed care	0.297	0.276	0.32	<0.0001	0.413	0.368	0.464	<0.0001	0.227	0.216	0.239	<0.0001
Medicaid	0.624	0.577	0.676	<0.0001	0.805	0.713	0.91	0.0005	0.584	0.552	0.619	<0.0001
Medicare	0.38	0.35	0.412	<0.0001	0.528	0.461	0.604	<0.0001	0.288	0.274	0.303	<0.0001
Other government	0.312	0.252	0.385	<0.0001	0.287	0.17	0.485	<0.0001	0.22	0.195	0.249	<0.0001
Year of diagnosis	0.89	0.876	0.903	<0.0001	0.937	0.91	0.964	<0.0001	0.899	0.893	0.906	<0.0001
Year of diagnosis—2009	1.106	1.08	1.133	<0.0001	1.034	0.988	1.082	0.1525	1.11	1.097	1.123	<0.0001
Odds ratio for presentation of metastatic NSCLC												
Facility type												
Community Cancer Program	–	–	–	–	–	–	–	–	–	–	–	–
Academic/Research Program	0.874	0.837	0.914	<0.0001	0.849	0.777	0.928	0.0003	0.829	0.817	0.84	<0.0001
Comprehensive Community Cancer Program	0.839	0.803	0.877	<0.0001	0.809	0.74	0.886	<0.0001	0.712	0.701	0.723	<0.0001
Integrated Network Cancer Program	0.88	0.836	0.926	<0.0001	0.956	0.861	1.062	0.4023	0.823	0.808	0.838	<0.0001
Facility location												
New England	–	–	–	–	–	–	–	–	–	–	–	–
Middle Atlantic	1.187	1.088	1.297	0.0001	1.046	0.916	1.194	0.504	1.044	1.023	1.066	<0.0001
South Atlantic	1.069	0.981	1.165	0.1293	0.98	0.859	1.119	0.7692	0.94	0.921	0.959	<0.0001
East North Central	1.11	1.017	1.211	0.0195	1.11	0.957	1.288	0.167	1.03	1.01	1.051	0.0037
East South Central	1.109	1.012	1.215	0.027	0.999	0.764	1.306	0.992	0.91	0.889	0.931	<0.0001
West North Central	1.089	0.985	1.204	0.0943	1.118	0.902	1.385	0.3099	1.044	1.02	1.068	0.0003
West South Central	1.153	1.054	1.262	0.0019	1.254	1.097	1.434	0.0009	0.985	0.962	1.009	0.2229
Mountain	1.193	1.021	1.394	0.026	1.152	0.989	1.341	0.0694	1.039	1.01	1.069	0.0078
Pacific	1.146	1.037	1.266	0.0074	1.251	1.097	1.427	0.0008	1.017	0.994	1.039	0.1474
Gender												
Male	–	–	–	–	–	–	–	–	–	–	–	–
Female	0.83	0.811	0.85	<0.0001	0.84	0.802	0.881	<0.0001	0.855	0.848	0.863	<0.0001
Age												
<50	–	–	–	–	–	–	–	–	–	–	–	–
50‐59	0.884	0.841	0.93	<0.0001	0.875	0.783	0.978	0.0185	0.859	0.838	0.88	<0.0001
60‐69	0.77	0.732	0.81	<0.0001	0.685	0.615	0.763	<0.0001	0.732	0.714	0.749	<0.0001
70+	0.712	0.675	0.751	<0.0001	0.647	0.579	0.722	<0.0001	0.669	0.653	0.686	<0.0001
Charlson comorbidity score												
0	–	–	–	–	–	–	–	–	–	–	–	–
1	0.834	0.812	0.856	<0.0001	0.797	0.754	0.841	<0.0001	0.766	0.758	0.774	<0.0001
2+	0.816	0.79	0.844	<0.0001	0.754	0.701	0.811	<0.0001	0.785	0.775	0.795	<0.0001
Demographic area												
Metropolitan	–	–	–	–	–	–	–	–	–	–	–	–
Rural	0.996	0.895	1.107	0.9376	1.158	0.774	1.732	0.4745	1	0.971	1.03	1
Urban	0.957	0.917	0.998	0.0405	1.006	0.905	1.119	0.9085	0.952	0.94	0.964	<0.0001
Income												
<$38 000	–	–	–	–	–	–	–	–	–	–	–	–
$38 000‐$47 999	0.991	0.961	1.023	0.5892	1.014	0.948	1.085	0.6829	1.018	1.003	1.033	0.0156
$48 000‐$62 999	0.987	0.95	1.026	0.5211	1.01	0.939	1.086	0.7938	1.034	1.018	1.051	<0.0001
$63 000+	1.014	0.963	1.067	0.6009	1.045	0.95	1.15	0.3659	1.043	1.024	1.062	<0.0001
Percent without high school diploma												
≥21%	–	–	–	–	–	–	–	–	–	–	–	–
13.0%‐20.9%	0.977	0.95	1.005	0.1089	0.941	0.882	1.005	0.0687	0.968	0.954	0.983	<0.0001
7.0%‐12.9%	0.947	0.909	0.986	0.0079	0.903	0.834	0.978	0.0119	0.962	0.946	0.977	<0.0001
<7.0%	0.906	0.852	0.964	0.0018	0.878	0.786	0.981	0.021	0.965	0.947	0.984	0.0003
Insurance status												
Uninsured	–	–	–	–	–	–	–	–	–	–	–	–
Private/managed care	0.696	0.66	0.733	<0.0001	0.578	0.524	0.638	<0.0001	0.604	0.588	0.621	<0.0001
Medicaid	0.849	0.803	0.898	<0.0001	0.75	0.673	0.835	<0.0001	0.733	0.71	0.758	<0.0001
Medicare	0.657	0.623	0.692	<0.0001	0.538	0.488	0.594	<0.0001	0.544	0.529	0.56	<0.0001
Other government	0.598	0.541	0.662	<0.0001	0.503	0.39	0.649	<0.0001	0.484	0.463	0.506	<0.0001
Year of diagnosis	0.983	0.975	0.992	0.0002	0.988	0.969	1.006	0.1962	0.969	0.966	0.972	<0.0001
Year of diagnosis—2009	1.029	1.015	1.043	<0.0001	1.012	0.984	1.04	0.4103	1.041	1.036	1.046	<0.0001
Odds ratio for presentation of metastatic prostate cancer												
Facility type												
Community Cancer Program	–	–	–	–	–	–	–	–	–	–	–	–
Academic/Research Program	0.871	0.81	0.94	0.0001	0.829	0.73	0.95	0.0054	0.797	0.77	0.82	<0.0001
Comprehensive Community Cancer Program	1.012	0.95	1.08	0.7283	0.987	0.87	1.12	0.8462	0.753	0.73	0.78	<0.0001
Integrated Network Cancer Program	0.993	0.92	1.08	0.8571	0.968	0.83	1.14	0.688	0.788	0.76	0.82	<0.0001
Facility location												
New England	–	–	–	–	–	–	–	–	–	–	–	–
Middle Atlantic	1.143	1.01	1.29	0.0304	1.056	0.87	1.28	0.5847	1.028	0.98	1.08	0.2277
South Atlantic	1.126	1	1.27	0.0519	1.191	0.98	1.46	0.0874	0.794	0.76	0.83	<0.0001
East North Central	1.108	0.98	1.25	0.101	1.189	0.96	1.48	0.1151	0.955	0.91	1	0.0365
East South Central	1.037	0.91	1.19	0.5914	1.385	0.89	2.15	0.1455	0.653	0.62	0.69	<0.0001
West North Central	1.229	1.06	1.43	0.0074	1.821	1.34	2.48	0.0001	1.023	0.97	1.08	0.358
West South Central	1.202	1.06	1.37	0.0054	1.689	1.39	2.05	<0.0001	0.886	0.84	0.94	<0.0001
Mountain	1.636	1.31	2.04	<0.0001	2.169	1.74	2.71	<0.0001	1.133	1.07	1.2	<0.0001
Pacific	1.476	1.28	1.7	<0.0001	1.589	1.32	1.92	<0.0001	0.979	0.94	1.03	0.375
Age												
<50	–	–	–	–	–	–	–	–	–	–	–	–
50‐59	1.017	0.92	1.13	0.7514	0.789	0.65	0.95	0.0136	0.741	0.69	0.8	<0.0001
60‐69	1.107	1	1.23	0.0515	0.689	0.57	0.83	<0.0001	0.723	0.67	0.78	<0.0001
70+	2.397	2.16	2.66	<0.0001	1.386	1.15	1.67	0.0006	1.856	1.73	2	<0.0001
Charlson comorbidity score												
0	–	–	–	–	–	–	–	–	–	–	–	–
1	0.977	0.93	1.03	0.3874	1.153	1.05	1.27	0.0027	1.229	1.2	1.26	<0.0001
2+	1.971	1.83	2.12	<0.0001	2.534	2.17	2.95	<0.0001	2.418	2.32	2.52	<0.0001
Demographic area												
Metropolitan	–	–	–	–	–	–	–	–	–	–	–	–
Rural	0.951	0.79	1.14	0.5811	0.847	0.45	1.6	0.6072	0.856	0.8	0.92	<0.0001
Urban	0.813	0.75	0.88	<0.0001	0.911	0.77	1.08	0.2699	0.863	0.84	0.89	<0.0001
Income												
<$38 000	–	–	–	–	–	–	–	–	–	–	–	–
$38 000‐$47 999	0.899	0.85	0.95	<0.0001	1.033	0.94	1.14	0.5139	0.926	0.89	0.96	<0.0001
$48 000‐$62 999	0.849	0.8	0.9	<0.0001	1.074	0.97	1.19	0.1866	0.911	0.88	0.95	<0.0001
$63 000+	0.715	0.66	0.78	<0.0001	0.998	0.87	1.15	0.9832	0.847	0.81	0.88	<0.0001
Percent without high school diploma												
≥21%	–	–	–	–	–	–	–	–	–	–	–	–
13.0%‐20.9%	0.974	0.93	1.02	0.2718	0.94	0.85	1.04	0.2094	0.884	0.85	0.92	<0.0001
7.0%‐12.9%	0.908	0.85	0.97	0.0051	0.846	0.75	0.96	0.0071	0.843	0.81	0.88	<0.0001
<7.0%	0.857	0.78	0.95	0.0022	0.762	0.65	0.9	0.0012	0.789	0.76	0.82	<0.0001
Insurance status												
Uninsured	–	–	–	–	–	–	–	–	–	–	–	–
Private/managed care	0.209	0.19	0.23	<0.0001	0.209	0.19	0.24	<0.0001	0.184	0.17	0.2	<0.0001
Medicaid	0.752	0.69	0.82	<0.0001	0.701	0.62	0.8	<0.0001	0.719	0.66	0.78	<0.0001
Medicare	0.329	0.31	0.36	<0.0001	0.321	0.29	0.36	<0.0001	0.247	0.23	0.26	<0.0001
Other government	0.145	0.12	0.17	<0.0001	0.219	0.15	0.32	<0.0001	0.181	0.16	0.2	<0.0001
Year of diagnosis	0.979	0.96	0.99	0.0048	1.014	0.99	1.04	0.3155	1.02	1.01	1.03	<0.0001
Year of diagnosis—2009	1.126	1.1	1.15	<0.0001	1.098	1.05	1.14	<0.0001	1.128	1.12	1.14	<0.0001

Note

The protective effect of insurance is noted specially for those holding private insurance and non‐Hispanic White patients.

We then performed a subgroup analysis for each cancer for impact of insurance status within each ethnicity. AA Patients with all forms of insurance continued to show significantly higher odds of presenting with metastatic cancers. Adjusting for region of facilities for initial presentation, odds of presentation with metastatic PCa for uninsured AA patients was OR 1.170 [95% CI, 1.1071‐1.291] compared to NHW patients. Surprisingly, this trend persists with AA who have private insurance and Medicare with OR 1.250 [95% CI, 1.199‐1.303] and 1.422 [95% CI, 1.378‐1.467] in PCa and BCa, respectively. Compared to uninsured NHW patients, uninsured H patients likewise have higher odds of presenting with metastatic PCa OR 1.158 [95% CI, 1.038‐1.291]. Similarly, the trend persists for H patients with Private insurance and Medicare, 1.215 [95% CI, 1.131‐1.305] and 1.159 [95% CI, 1.098‐1.224]. Uninsured AA patients with BCa have OR 1.131 [95% CI, 1.043‐1.227] compared to uninsured NHW. AA patients with private insurance and Medicare have OR 1.450 [95% CI, 1.399‐1.503] and 1.458 [95% CI, 1.407‐1.510] for PCa and BCa, respectively. Interestingly, uninsured AA patients have similar odds of presenting with metastatic NSCLC compared to NHW patients. However, uninsured Hispanic patients have higher odds of presenting with stage IV NSCLC OR 1.296 [95% CI, 1.185‐1.418]. A summary of these findings is presented in Tables 2. Finally, the analysis of effect revealed insurance status to be a significant predictor of stage IV diagnosis within each ethnicity and for each cancer type (Table S2).

**Table 2:** Hispanic and African‐American patients have greater odds of presenting with metastatic Breast Cancer, NSCLC, and Prostate Cancer regardless of insurance level or cancer type

Baseline characteristics	Estimate	95% Confidence limits		*P*‐value
Logistic regression with interaction between race (Non‐Hispanic White, African American, Hispanic, and Other) and insurance for breast cancer				
Odds Ratio for metastatic breast cancer				
Facility type				
Community cancer program	–	–	–	–
Academic/research program	0.995	0.968	1.022	0.7015
Comprehensive community cancer program	0.905	0.882	0.929	<0.0001
Integrated network cancer program	0.983	0.951	1.016	0.3131
Facility location				
New England	–	–	–	–
Middle Atlantic	1.155	1.110	1.202	<0.0001
South Atlantic	1.066	1.015	1.118	0.0099
East North Central	1.289	1.238	1.342	<0.0001
East South Central	1.080	1.026	1.138	0.0035
West North Central	1.015	0.973	1.059	0.4911
West South Central	1.076	1.034	1.120	0.0003
Mountain	1.123	1.071	1.177	<0.0001
Pacific	1.076	1.028	1.126	0.0017
Race				
African American vs Non‐Hispanic White (Uninsured)	1.131	1.043	1.227	0.0029
Hispanic vs Non‐Hispanic White (Uninsured)	0.562	0.507	0.624	<0.0001
Other vs Non‐Hispanic White (Uninsured)	0.695	0.598	0.809	<0.0001
African American vs Non‐Hispanic White (Private/managed care)	1.450	1.399	1.503	<0.0001
Hispanic vs Non‐Hispanic White (Private/managed care)	0.959	0.900	1.022	0.1974
Other vs Non‐Hispanic White (Private/managed care)	0.893	0.837	0.953	0.0007
African American vs Non‐Hispanic White (Medicaid)	1.157	1.095	1.223	<0.0001
Hispanic vs Non‐Hispanic White (Medicaid)	0.706	0.653	0.763	<0.0001
Other vs Non‐Hispanic White (Medicaid)	0.794	0.717	0.879	<0.0001
African American vs Non‐Hispanic White (Medicare)	1.458	1.407	1.510	<0.0001
Hispanic vs Non‐Hispanic White (Medicare)	1.017	0.950	1.090	0.6246
Other vs Non‐Hispanic White (Medicare)	1.011	0.934	1.094	0.7908
African American vs Non‐Hispanic White (Other government)	1.654	1.314	2.083	<0.0001
Hispanic vs Non‐Hispanic White (Other government)	0.721	0.425	1.223	0.2256
Other vs Non‐Hispanic White (Other government)	1.662	1.246	2.217	0.0006
Age				
<50	–	–	–	–
50‐59	1.268	1.235	1.301	<0.0001
60‐69	1.220	1.187	1.254	<0.0001
70+	1.286	1.246	1.327	<0.0001
Charlson comorbidity score				
0	–	–	–	–
1	1.074	1.050	1.098	<0.0001
2+	1.589	1.533	1.647	<0.0001
Demographic area				
Metropolitan	–	–	–	–
Rural	0.973	0.911	1.039	0.4123
Urban	0.937	0.913	0.962	<0.0001
Income				
<$38 000	–	–	–	–
$38 000‐$47 999	0.975	0.949	1.001	0.0550
$48 000‐$62 999	0.969	0.942	0.997	0.0304
$63 000+	0.966	0.935	0.998	0.0387
Percent without high school diploma				
≥21%	–	–	–	–
13.0%‐20.9%	0.926	0.902	0.949	<0.0001
7.0%‐12.9%	0.873	0.848	0.898	<0.0001
<7.0%	0.753	0.727	0.779	<0.0001
Insurance status				
Private/managed care vs Uninsured (African American)	0.292	0.272	0.315	<0.0001
Medicaid vs Uninsured (African American)	0.603	0.557	0.653	<0.0001
Medicare vs Uninsured (African American)	0.372	0.345	0.401	<0.0001
Other Government vs Uninsured (African American)	0.319	0.258	0.394	<0.0001
Private/managed care vs Uninsured (Hispanic)	0.389	0.348	0.435	<0.0001
Medicaid vs Uninsured (Hispanic)	0.740	0.659	0.832	<0.0001
Medicare vs Uninsured (Hispanic)	0.522	0.465	0.586	<0.0001
Other government vs Uninsured (Hispanic)	0.280	0.166	0.472	<0.0001
Private/managed care vs Uninsured (Other)	0.293	0.251	0.343	<0.0001
Medicaid vs Uninsured (Other)	0.673	0.567	0.800	<0.0001
Medicare vs Uninsured (Other)	0.419	0.356	0.494	<0.0001
Other government vs Uninsured (Other)	0.521	0.386	0.705	<0.0001
Private/managed care vs Uninsured (Non‐Hispanic White)	0.228	0.217	0.239	<0.0001
Medicaid vs Uninsured (Non‐Hispanic White)	0.590	0.557	0.624	<0.0001
Medicare vs Uninsured (Non‐Hispanic White)	0.289	0.274	0.304	<0.0001
Other government vs Uninsured (Non‐Hispanic White)	0.218	0.193	0.246	<0.0001
Year of diagnosis	0.899	0.894	0.905	<0.0001
Year of diagnosis—2009	1.106	1.095	1.117	<0.001
Logistic regression with interaction between race (Non‐Hispanic Whites, African American, Hispanic, and Other) and insurance for NSCLC				
Odds ratio for metastatic NSCLC				
Facility type				
Community Cancer Program				
Academic/Research Program	0.835	0.824	0.846	<0.0001
Comprehensive Community Cancer Program	0.730	0.720	0.740	<0.0001
Integrated Network Cancer Program	0.832	0.818	0.846	<0.0001
Facility location				
New England	–	–	–	–
Middle Atlantic	1.044	1.024	1.064	<0.0001
South Atlantic	0.942	0.924	0.960	<0.0001
East North Central	1.029	1.010	1.049	0.0029
East South Central	0.921	0.901	0.942	<0.0001
West North Central	1.042	1.020	1.065	0.0002
West South Central	1.005	0.983	1.027	0.6608
Mountain	1.046	1.019	1.075	0.0009
Pacific	1.033	1.012	1.055	0.0021
Gender				
Male	–	–	–	–
Female	0.856	0.849	0.863	<0.0001
Race				
African American vs Non‐Hispanic White (Uninsured)	1.006	0.953	1.061	0.8297
Hispanic vs Non‐Hispanic White (Uninsured)	1.296	1.185	1.418	<0.0001
Other vs Non‐Hispanic White (Uninsured)	1.382	1.237	1.544	<0.0001
African American vs Non‐Hispanic White (Private/managed care)	1.140	1.112	1.168	<0.0001
Hispanic vs Non‐Hispanic White (Private/managed care)	1.181	1.127	1.238	<0.0001
Other vs Non‐Hispanic White (Private/managed care)	1.233	1.185	1.283	<0.0001
African American vs Non‐Hispanic White (Medicaid)	1.160	1.119	1.203	<0.0001
Hispanic vs Non‐Hispanic White (Medicaid)	1.282	1.202	1.368	<0.0001
Other vs Non‐Hispanic White (Medicaid)	1.239	1.157	1.325	<0.0001
African American vs Non‐Hispanic White (Medicare)	1.224	1.203	1.245	<0.0001
Hispanic vs Non‐Hispanic White (Medicare)	1.204	1.165	1.244	<0.0001
Other vs Non‐Hispanic White (Medicare)	1.247	1.208	1.287	<0.0001
African American vs Non‐Hispanic White (Other government)	1.265	1.150	1.392	<0.0001
Hispanic vs Non‐Hispanic White (Other government)	1.298	1.021	1.651	0.0335
Other vs Non‐Hispanic White (Other government)	1.186	1.019	1.381	0.0275
Age				
<50	–	–	–	–
50‐59	0.859	0.841	0.877	<0.0001
60‐69	0.731	0.716	0.746	<0.0001
70+	0.671	0.657	0.685	<0.0001
Charlson comorbidity score				
0	–	–	–	–
1	0.775	0.768	0.782	<0.0001
2+	0.788	0.778	0.797	<0.0001
Demographic area				
Metropolitan				
Rural	0.992	0.965	1.020	0.5755
Urban	0.949	0.938	0.960	<0.0001
Income				
<$38 000	–	–	–	–
$38 000‐$47 999	1.008	0.995	1.021	0.2228
$48 000‐$62 999	1.023	1.008	1.037	0.0016
$63 000+	1.032	1.015	1.048	0.0002
Percent without high school diploma				
≥21%				
13.0%‐20.9%	0.966	0.954	0.978	<0.0001
7.0%‐12.9%	0.957	0.943	0.970	<0.0001
<7.0%	0.959	0.943	0.976	<0.0001
Insurance status				
Private/managed care vs Uninsured (African American)	0.687	0.652	0.723	<0.0001
Medicaid vs Uninsured (African American)	0.845	0.8	0.894	<0.0001
Medicare vs Uninsured (African American)	0.664	0.631	0.697	<0.0001
Other Government vs Uninsured (African American)	0.608	0.55	0.672	<0.0001
Private/managed care vs Uninsured (Hispanic)	0.552	0.501	0.609	<0.0001
Medicaid vs Uninsured (Hispanic)	0.725	0.652	0.805	<0.0001
Medicare vs Uninsured (Hispanic)	0.506	0.462	0.555	<0.0001
Other government vs Uninsured (Hispanic)	0.484	0.376	0.623	<0.0001
Private/managed care vs Uninsured (Other)	0.541	0.483	0.607	<0.0001
Medicaid vs Uninsured (Other)	0.657	0.579	0.745	<0.0001
Medicare vs Uninsured (Other)	0.492	0.44	0.551	<0.0001
Other government vs Uninsured (Other)	0.415	0.345	0.498	<0.0001
Private/managed care vs Uninsured (Non‐Hispanic White)	0.606	0.59	0.623	<0.0001
Medicaid vs Uninsured (Non‐Hispanic White)	0.733	0.709	0.757	<0.0001
Medicare vs Uninsured (Non‐Hispanic White)	0.545	0.53	0.561	<0.0001
Other government vs Uninsured (Non‐Hispanic White)	0.483	0.462	0.505	<0.0001
Year of diagnosis	0.972	0.969	0.975	<0.0001
Year of diagnosis‐2009	1.038	1.034	1.043	<0.0001
Logistic regression with interaction between race (Non‐Hispanic Whites, African American, Hispanic, and Other) and insurance for prostate cancer				
Odds ratio for metastatic prostate cancer odds ratio for metastatic prostate cancer				
Facility type				
Community Cancer Program	–	–	–	–
Academic/Research Program	0.815	0.793	0.839	<0.0001
Comprehensive Community Cancer Program	0.81	0.787	0.834	<0.0001
Integrated Network Cancer Program	0.834	0.804	0.865	<0.0001
Facility location				
New England				
Middle Atlantic	1.021	0.981	1.063	0.3096
South Atlantic	0.85	0.818	0.885	<0.0001
East North Central	0.983	0.945	1.023	0.3913
East South Central	0.729	0.694	0.766	<0.0001
West North Central	1.076	1.028	1.126	0.0016
West South Central	0.973	0.928	1.02	0.2565
Mountain	1.237	1.175	1.302	<0.0001
Pacific	1.063	1.02	1.108	0.004
Race				
African American vs Non‐Hispanic White (Uninsured)	1.17	1.071	1.278	0.0005
Hispanic vs Non‐Hispanic White (Uninsured)	1.158	1.038	1.291	0.0084
Other vs Non‐Hispanic White (Uninsured)	0.955	0.794	1.15	0.6277
African American vs Non‐Hispanic White (Private/managed care)	1.25	1.199	1.303	<0.0001
Hispanic vs Non‐Hispanic White (Private/managed care)	1.215	1.131	1.305	<0.0001
Other vs Non‐Hispanic White (Private/managed care)	1.052	0.967	1.145	0.24
African American vs Non‐Hispanic White (Medicaid)	1.097	1.017	1.184	0.0171
Hispanic vs Non‐Hispanic White (Medicaid)	0.893	0.806	0.989	0.0293
Other vs Non‐Hispanic White (Medicaid)	0.768	0.666	0.884	0.0003
African American vs Non‐Hispanic White (Medicare)	1.422	1.378	1.467	<0.0001
Hispanic vs Non‐Hispanic White (Medicare)	1.159	1.098	1.224	<0.0001
Other vs Non‐Hispanic White (Medicare)	1.051	0.984	1.121	0.1371
African American vs Non‐Hispanic White (Other government)	0.913	0.762	1.095	0.3265
Hispanic vs Non‐Hispanic White (Other government)	1.373	0.947	1.99	0.0943
Other vs Non‐Hispanic White (Other government)	1.924	1.402	2.64	<0.0001
Age				
<50	–	–	–	–
50‐59	0.829	0.784	0.877	<0.0001
60‐69	0.822	0.777	0.868	<0.0001
70+	1.992	1.882	2.107	<0.0001
Charlson comorbidity score				
0	–	–	–	–
1	1.167	1.14	1.194	<0.0001
2+	2.317	2.238	2.399	<0.0001
Demographic area				
Metropolitan	–	–	–	–
Rural	0.871	0.819	0.927	<0.0001
Urban	0.865	0.842	0.888	<0.0001
Income				
<$38 000	–	–	–	–
$38 000‐$47 999	0.923	0.898	0.949	<0.0001
$48 000‐$62 999	0.902	0.876	0.929	<0.0001
$63 000+	0.828	0.8	0.857	<0.0001
Percent without high school diploma				
≥21%	–	–	–	–
13.0%‐20.9%	0.926	0.901	0.951	<0.0001
7.0%‐12.9%	0.873	0.847	0.9	<0.0001
<7.0%	0.819	0.79	0.849	<0.0001
Insurance status				
Private/managed care vs Uninsured (African American)	0.199	0.185	0.214	<0.0001
Medicaid vs Uninsured (African American)	0.695	0.64	0.756	<0.0001
Medicare vs Uninsured (African American)	0.313	0.291	0.336	<0.0001
Other Government vs Uninsured (African American)	0.143	0.12	0.169	<0.0001
Private/managed care vs Uninsured (Hispanic)	0.195	0.174	0.219	<0.0001
Medicaid vs Uninsured (Hispanic)	0.572	0.505	0.648	<0.0001
Medicare vs Uninsured (Hispanic)	0.258	0.232	0.286	<0.0001
Other government vs Uninsured (Hispanic)	0.217	0.149	0.314	<0.0001
Private/managed care vs Uninsured (Other)	0.205	0.169	0.249	<0.0001
Medicaid vs Uninsured (Other)	0.596	0.479	0.741	<0.0001
Medicare vs Uninsured (Other)	0.283	0.235	0.341	<0.0001
Other government vs Uninsured (Other)	0.368	0.259	0.522	<0.0001
Private/managed care vs Uninsured (Non‐Hispanic White)	0.186	0.175	0.198	<0.0001
Medicaid vs Uninsured (Non‐Hispanic White)	0.741	0.684	0.804	<0.0001
Medicare vs Uninsured (Non‐Hispanic White)	0.257	0.242	0.274	<0.0001
Other government vs Uninsured (Non‐Hispanic White)	0.183	0.164	0.203	<0.0001
Year of diagnosis	1.012	1.005	1.019	0.0004
Year of diagnosis‐2009	1.125	1.114	1.136	<0.0001

Note

Patients were stratified based on ethnicity and insurance status. A multilevel logistic regression was conducted to determine the role of holding the same insurance for different ethnicities within all 3 cancer types.

## 4 DISCUSSION

In this analysis of the National Cancer Database, we have consistently identified the effect of insurance status in predicting metastatic presentation in three common cancer types (breast, prostate and non–small cell lung cancer). Additionally, insurance status and ethnicity affect the odds of presenting with metastatic cancer in all three sites. Finally, the degree of advantage conferred by insurance status appears to differ across ethnicities. A decade ago, a landmark study addressed this very question in multiple cancers using the NCDB.^16^ Our results, using a subset comprised of fewer cancer types, mirror those findings and, significantly, reveal a persistent trend reflecting disparity despite comparable insurance status that exists across years and disease sites.

Our findings suggest that insurance coverage is a powerful protective factor against metastatic cancer presentation. Access to healthcare can be a critical barrier for cancer patients and insurance status has previously been linked to patients presenting with more advanced disease across different cancer types.^16^ In our study, Medicaid recipients had decreased odds of metastatic presentation in all three cancers compared to uninsured patients but consistently lagged patients with private insurance. This is in line with the fact that Medicaid recipients typically have access to inferior care compared to those with private insurance.^17^, ^18^ This may partially leads to poorer quality of care for minority patients, given that a higher proportion of these patients receive Medicaid.^19^

Moreover, minority patients, who historically have had worse cancer outcomes due to a multitude of factors including limited or inferior access to health services, appear to not benefit as much from insurance access as NHW patients even adjusting for available demographic features. It is therefore imperative that any effort to address cancer care disparity attempts to understand the factors which impede access to early detection in minority populations aside from insurance.

Minority patients’ patterns of cancer screening utilization and preventative health behaviors differ from that of NHW patients both in frequency of access and quality of care.^20^, ^21^ Historically, minority patients have not utilized mammography at the same rate as their NHW counterparts^22^ or undergone “high‐quality” mammograms.^23^ Likewise, in prostate cancer, AA men undergo less‐frequent PSA screening – a critical difference as they are 70% more likely to be diagnosed with prostate cancer and have a PCa mortality rate 2.4 times that of NHW.^24^ Compounding missed opportunities for early detection and timely intervention are systemic disparities, including that minority‐serving hospitals often lack dedicated cancer screening programs with expertise and technologies comparable to the academic centers serving more affluent populations.^25^

Recent evidence has shown minority patients’ care differs even within the same hospital compared to NHW patients.^26^, ^27^, ^28^ Pain and neurological symptoms are more commonly overlooked in AA and H patients, a lapse that may lead to dismissal of nonspecific symptoms that could warrant further workup.^29^, ^30^ Our results complement similar studies showing, despite the same insurance, disparities along ethnic lines exist among patients whether in rates of metastatic presentation or treatment utilization. Attitude of health‐care providers toward minority patients,^31^ deficiencies in cancer prevention in minority serving hospitals, and possible discrepancy in health literacy in minority populations^32^ can be additional confounders in metastatic cancer presentations.

We included NSCLC, a site with significant risk of metastasis at presentation, which differs from the other two cancer types in that screening tests are in their infancy and not widely utilized. While low‐dose CT screening is promising,^33^ a majority of patients present with metastatic disease in the NCDB, likely due to pathophysiology of disease overwhelming other demographic features. However, non‐White patients consistently continue to have a higher risk of presenting with metastatic cancers. Interestingly, private insurance offers the smallest risk reduction against metastatic presentation of NSCLC for all ethnic groups.

Significant efforts have been undertaken to define biological determinant of health‐care disparity. However, these biologic factors cannot fully explain the rates of metastatic presentation in various ethnicities.^34^, ^35^ For example, AA patients experience different odds of metastatic prostate cancer presentation in different regions (Table 1 and Figure S2). It is unlikely that a unique underlying biological driver can account for geographic disparity.

The disproportionate rates of metastatic presentation in minority patients suggest that barriers to early detection are rooted in a complex set of factors related to region and quality of insurance. Protective effects of insurance are tempered by structural obstacles facing minority patients, and providing insurance, albeit a critical step, may not sufficiently alter odds of metastatic presentation without addressing barriers to early detection, quality primary and oncologic care, and improved health literacy.

## 5 STRENGTHS AND LIMITATION

The strength of this analysis lies in the use of a large, curated database serving a diverse cohort of patients representing the majority of cancer diagnosis in the US population. The NCDB enables direct examination of the role of various insurance types on specific cancer presentation accounting for patient‐level and sociodemographic features. Two significant developments highlight NCDB’s usefulness since the prior publication addressing issues of insurance and ethnicity came out a decade ago. Since 2009, CoC accredited centers were required to report metastatic presentation in patients at the time of diagnosis. Additionally, with increasing participation of minorities in diverse geographical regions, a more wholly representative demographic is captured.^36^, ^37^

Nevertheless, this analysis has limitations inherent to any retrospective database research. Several unmeasured confounders are present. There are significant differences in the experience of Hispanic and African American patients in the health‐care system. Language barriers, institutional biases, and immigration status can define the experiences of some while not pose a barrier to others. Additionally, self‐reported ethnicity could be difficult to capture in such large datasets. African‐American ethnicity may be reported adequately while Hispanic patients may be misrepresented. Additionally, while zip code–level data rarely provide a complete view of a single patient's social setting, in this and many other reports using NCDB, the census‐based demographics have been used to draw conclusions regarding individual‐level care. This cannot fully capture the complex interaction of patients with social determinant of their care within their community. Finally, metastatic cancer manifests in a spectrum of low to high burden disease. This inevitably affects the patient's utilization of health services. The current report cannot sufficiently characterize and adjust for different modes of presentations. Furthermore, the NCDB itself has certain drawbacks. Although diverse facility types and regions are represented in the NCDB, not all hospitals are CoC accredited. Therefore, depending on the demographics of nonaccredited centers, the conclusions may slightly differ.

## 6 CONCLUSION

Metastatic cancer presentation is a significant challenge for all patients and uniquely burdensome for the uninsured. Although protective effects of insurance are undeniable, as evidenced in our analysis, this effect is modulated by ethnicity, and quality of insurance and even within a given insurance, certain populations are more vulnerable to metastatic cancer presentation. Recent policy changes provide the opportunity to study the effect of insurance access before and after expansion of various state‐ and federal‐level programs. This is a critical mandate as we aim to prevent late cancer diagnoses in at‐risk populations. Finally, future studies using similar databases may address the role of timely therapeutic interventions for metastatic patients and their effect in closing the disparity gap

## CONFLICT OF INTEREST

The Authors declare no conflict of interest.

## Author contributions

Conception and design: NA and KU. Data analysis and interpretation: HW, MM, SF, and NA. Manuscript writing: All authors. Final approval of manuscript: All authors.

## Supporting information

Figure S1

Table S1

Table S2
