# Supplementary figures and images for: Ethnicity and insurance status predict metastatic disease presentation in prostate, breast, and non‐small cell lung cancer
Source: Cancer Med. 2020 Jun 8;9(15):5362–80. doi: 10.1002/cam4.3109 (PMC7402826; doi:10.1002/cam4.3109)

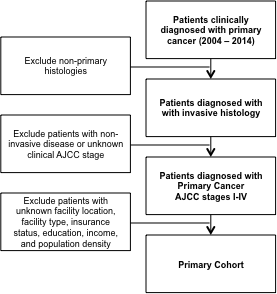


The same CONSORT diagram applies for breast, prostate, and lung cancer.

Supplement: Supplementary file 1 — Figure S1 [file CAM4-9-5362-s001.docx]
