# Supplementary material for: Ethnicity and insurance status predict metastatic disease presentation in prostate, breast, and non‐small cell lung cancer
Source: Cancer Med. 2020 Jun 8;9(15):5362–80. doi: 10.1002/cam4.3109 (PMC7402826; doi:10.1002/cam4.3109)
Supplement: Supplementary file 2 — Table S1 [file CAM4-9-5362-s002.docx]

Supplementary Table 1.Bivariate analysis of baseline characteristics for breast cancer patients stratified on AJCC stage at diagnosis.

Table 1.1: Bivariate analysis of baseline characteristics for breast cancer patients stratified on AJCC stage at diagnosis

| Baseline characteristics | Stage I-III (%)  (1199270) | Stage IV (%)  (80322) | P-value |
| --- | --- | --- | --- |
| Facility type  Community Cancer Program  Comprehensive Community Cancer Program  Academic/Research Program  Integrated Network Cancer Program | 127747 (11.20)  550983 (48.32)  340532 (29.86)  121020 (10.61) | 9329 (12.33)  33426 (44.19)  24740 (32.71)  8144 (10.77) | <0.0001 |
| Facility location  New England  Middle Atlantic  South Atlantic  East North Central  East South Central  West North Central  West South Central  Mountain  Pacific | 72866 (6.39)  172467 (15.12)  243824 (21.38)  208623 (18.30)  67714 (5.94)  82788 (7.26)  90010 (7.89)  55373 (7.86)  146617 (12.85) | 4046 (5.31)  12941 (17.11)  16505 (21.82)  14585 (19.28)  4590 (6.07)  5007 (6.62)  6412 (8.48)  8364 (11.06)  75639 (6.22) | <0.0001 |
| Gender  Male  Female | 11238 (0.94)  1188032 (99.06) | 1113 (1.39)  79209 (98.61) | <0.0001 |
| Age  <50  50-59  60-69  70+ | 255314 (21.29)  289105 (24.11)  310512 (25.89)  344339 (28.71) | 15600 (19.42)  19855 (24.72)  20311 (25.29)  24556 (30.57) | <0.0001 |
| Charlson comorbidity score  0  1  2+ | 1008790 (84.12)  154532 (12.89)  35948 (3.00) | 64789 (80.66)  11378 (14.17)  4155 (5.17) | <0.0001 |
| Demographic area  Metropolitan  Urban  Rural | 1006809 (86.49)  140097 (12.03)  17209 (1.48) | 67074 (86.44)  9318 (12.01)  1208 (1.56) | 0.2142 |
| Income  <$38,000  $38,000-$47999  $48000-$62999  $63000+ | 176320 (14.82)  252453 (21.22)  318283 (26.75)  442714 (37.21) | 15611 (19.75)  18154 (22.97)  20742 (26.24)  24537 (31.04) | <0.0001 |
| Percent without high school diploma  21%+  13.0-20.9%  7.0-12.9%  <7.0% | 172702 (14.51)  282026 (23.69)  393781 (33.08)  341756 (28.71) | 15253 (19.29)  20989 (26.54)  25292 (31.98)  17551 (22.19) | <0.0001 |
| Insurance status  Not insured  Private insurance/managed care  Medicaid  Medicare  Other government | 24017 (2.04)  615504 (52.32)  72244 (6.14)  453436 (38.55)  11135 (0.95) | 4735 (6.07)  31456 (40.29)  9372 (12.00)  31910 (40.87)  597 (0.76) | <0.0001 |
| Year of diagnosis  2004  2005  2006  2007  2008  2009  2010  2011  2012  2013  2014 | 49027 (4.09)  52989 (4.42)  57661 (4.81)  70451 (5.87)  108518 (9.05)  126193 (10.52)  133717 (11.15)  142223 (11.86)  147159 (12.47)  154672 (12.90)  156660 (13.06) | 4762 (5.93)  4862 (6.05) 5131 (6.39)  5787 (7.20)  7132 (8.88)  7765 (9.67)  8140 (10.13)  8716 (10.85)  8997 (11.20)  9356 (11.65)  9674 (12.04) | <0.0001 |

Table 1.2: Bivariate analysis of baseline characteristics for prostate cancer patients stratified on AJCC stage at diagnosis

| Baseline characteristics | Stage I-III (%)  (1122785) | Stage IV (%)  (68414) | P-value |
| --- | --- | --- | --- |
| Facility type  Community Cancer Program  Comprehensive Community Cancer Program  Academic/Research Program  Integrated Network Cancer Program | 95380 (8.50)  487682 (43.46)  424359 (37.82)  114679 (10.22) | 7839 (11.47)  28862 (42.22)  24844 (36.34)  6817 (9.97) | <0.0001 |
| Facility location  New England  Middle Atlantic  South Atlantic  East North Central  East South Central  West North Central  West South Central  Mountain  Pacific | 71697 (6.39)  176203 (15.70)  246716 (21.99)  200870 (17.90)  79442 (7.08)  93716 (8.35)  72108 (6.43)  47266 (4.21)  134082 (11.95) | 4334 (6.34)  10797 (15.79)  13828 (20.23)  12848 (18.79)  3938 (8.76)  5564 (8.14)  5329 (7.80)  3239 (4.74)  8485 (12.41) | <0.0001 |
| Gender  Male | 1122785 (100%) | 68414 (100%) | - |
| Age  <50  50-59  60-69  70+ | 36699 (3.27)  268372 (23.90)  477854 (42.56)  339860 (30.27) | 10948 (16.00)  20277 (29.64)  35419 (51.77)  1770 (2.59) | <0.0001 |
| Age (continuous), Mean (SD) | 65.04 (8.74) | 70.31 (11.18) | <0.0001 |
| Charlson comorbidity score  0  1  2+ | 947603 (84.40)  147917 (13.17)  27265 (2.43) | 53126 (77.65)  10623 (15.53)  4665 (6.82) | <0.0001 |
| Demographic area  Metropolitan  Urban  Rural | 912040 (83.55)  15762 (14.44)  21946 (2.01) | 55646 (83.96)  9295 (14.02)  1335 (2.01) | 0.0118 |
| Income  <$38,000  $38,000-$47999  $48000-$62999  $63000+ | 173816 (15.60)  240897 (21.62)  296418 (26.60)  403048 (36.17) | 13956 (20.67)  15697 (23.24)  17888 (26.49)  19993 (29.60) | <0.0001 |
| Percent without high school diploma  21%+  13.0-20.9%  7.0-12.9%  <7.0% | 163852 (14.70)  266032 (23.86)  367996 (33.01)  316941 (28.43) | 13552 (20.05)  17521 (25.92)  20992 (31.06)  15523 (22.97) | <0.0001 |
| Insurance status  Not insured  Private insurance/managed care  Medicaid  Medicare  Other government | 16847 (1.53)  530247 (48.24)  25681 (2.34)  506153 (46.04)  20363 (1.85) | 3469 (5.22)  18249 (27.46)  4445 (6.69)  39481 (59.41)  815 (1.23) | <0.0001 |
| Year of diagnosis  2004  2005  2006  2007  2008  2009  2010  2011  2012  2013  2014  2015 | 79216 (7.06)  81378 (7.25)  90844 (8.09)  100879 (8.98)  107322 (9.56)  106316 (9.47)  103733 (9.24)  106398 (9.48)  88483 (7.88)  87059 (7.75)  83213 (7.41)  87944 (7.83) | 3889 (5.68)  4146 (6.06)  4248 (6.21)  4396 (6.43)  4908 (7.17)  5316 (7.77)  5478 (8.01)  5787 (8.46)  6245 (9.13)  7181 (10.50)  7910 (11.56)  8910 (13.02) | <0.0001 |

.

Table 1.3: Bivariate analysis of baseline characteristics for lung cancer patients stratified on AJCC stage at diagnosis

| Baseline characteristics | Stage I-III (%)  (668225) | Stage IV (%)  (513221) | P-value |
| --- | --- | --- | --- |
| Facility type  Community Cancer Program  Comprehensive Community Cancer Program  Academic/Research Program  Integrated Network Cancer Program | 66934 (10.07)  310528 (46.70)  216748 (32.60)  70732 (10.64) | 61609 912.09)  237389 (46.60)  156286 (30.68)  54093 (10.62) | <0.0001 |
| Facility location  New England  Middle Atlantic  South Atlantic  East North Central  East South Central  West North Central  West South Central  Mountain  Pacific | 40568 (6.10)  97844 (14.71)  152753 (22.97)  126634 (19.04)  58789 (8.84)  53268 (8.01)  50030 (7.52)  22746 (3.42)  62310 (9.37) | 30198 (5.93)  75820 (14.88)  112665 (22.12)  98931 (19.42)  41682 (8.18)  40565 (7.96)  40375 (7.93)  18030 (3.54)  51111 (10.03) | <0.0001 |
| Gender  Male  Female | 344105 (51.50)  324120 (48.50) | 283256 (55.19)  229965 (44.81) | <0.0001 |
| Age  <50  50-59  60-69  70+ | 26506 (3.97)  97195 (14.55)  200127 (29.95)  344397 (51.54) | 32317 (6.30)  97645 (19.03)  154241 (30.05)  229018 (44.62) | <0.0001 |
| Age (continuous), Mean (SD), Min, Max | 69.14 (10.52) | 67.34 (11.30) | <0.0001 |
| Charlson comorbidity score  0  1  2+ | 373936 (55.96)  201238 (30.12)  93051 (13.93) | 321799 (62.70)  130997 (25.52)  60425 (11.77) | <0.0001 |
| Demographic area  Metropolitan  Urban  Rural | 526540 (81.46)  105372 (16.30)  14471 (2.24) | 405706 (82.05)  77790 (15.730  10948 (2.21) | <0.0001 |
| Income  <$38,000  $38,000-$47999  $48000-$62999  $63000+ | 132891 (20.17)  167181 (25.37)  176622 (26.80)  182323 (27.67) | 104552 (20.76)  127017 (25.22)  134499 (26.70)  137581 (27.32) | <0.0001 |
| Percent without high school diploma  21%+  13.0-20.9%  7.0-12.9%  <7.0% | 117031 (17.75)  187293 928.41)  218875 (33.20)  136120 (20.65) | 95414 (18.93)  143202 928.42)  164124 (32.57)  101173 (20.08) | <0.0001 |
| Insurance Status  Not insured  Private insurance/managed care  Medicaid  Medicare  Other government | 15310 (2.33)  167660 (25.57)  34971 (5.33)  427198 (65.15)  10593 (1.62) | 22532 (4.49)  145830 (29.05)  38475 (7.66)  288235 (57.41)  6994 (1.39) | <0.0001 |
| Year of diagnosis  2004  2005  2006  2007  2008  2009  2010  2011  2012  2013  2014  2015 | 37839 (5.66)  40246 (6.02)  40612 (6.08)  43497 (6.51)  55841 (8.36)  61024 (9.13)  60860 (9.11)  61761 (9.24)  63488 (5.37)  65838 (9.85)  6775 (10.14)  69444 (10.39) | 32651 (6.36)  33661 (6.56)  34265 (6.68)  35306 (6.88)  40177 (7.83)  42507 (8.28)  47297 (9.22)  47246 (9.21)  48648 (9.48)  50368 (9.81)  50854 (9.91)  50241 (9.79) | <0.0001 |
