# Supplementary material for: Ethnicity and insurance status predict metastatic disease presentation in prostate, breast, and non‐small cell lung cancer
Source: Cancer Med. 2020 Jun 8;9(15):5362–80. doi: 10.1002/cam4.3109 (PMC7402826; doi:10.1002/cam4.3109)
Supplement: Supplementary file 3 — Table S2 [file CAM4-9-5362-s003.docx]

| Type 3 Analysis of Effects | | | |
| --- | --- | --- | --- |
| Effect | DF | Wald Chi-Square | Pr > ChiSq |
| FACILITY_TYPE_CD | 3 | 63.2411 | <.0001 |
| FACILITY_LOCATION_CD | 8 | 44.8213 | <.0001 |
| SEX | 1 | 252.6582 | <.0001 |
| age | 3 | 221.7604 | <.0001 |
| CDCC_TOTAL_BEST | 2 | 256.0854 | <.0001 |
| UR_CD_13 | 2 | 4.2057 | 0.1221 |
| MED_INC_QUAR_12 | 3 | 1.5326 | 0.6748 |
| NO_HSD_QUAR_12 | 3 | 11.5478 | 0.0091 |
| INSURANCE_STATUS | 4 | 363.5798 | <.0001 |
| YEAR_OF_DIAGNOSIS | 1 | 13.7547 | 0.0002 |
| YEAR_OF_DIAGNOSIS-2009 | 1 | 17.0177 | <.0001 |

Supplementary table 2.1 NSCLC African American patients

| Type 3 Analysis of Effects | | | |
| --- | --- | --- | --- |
| Effect | DF | Wald Chi-Square | Pr > ChiSq |
| FACILITY_TYPE_CD | 3 | 33.1172 | <.0001 |
| FACILITY_LOCATION_CD | 8 | 62.4323 | <.0001 |
| SEX | 1 | 52.2237 | <.0001 |
| age | 3 | 99.2899 | <.0001 |
| CDCC_TOTAL_BEST | 2 | 99.2199 | <.0001 |
| UR_CD_13 | 2 | 0.5187 | 0.7716 |
| MED_INC_QUAR_12 | 3 | 0.9575 | 0.8115 |
| NO_HSD_QUAR_12 | 3 | 7.9873 | 0.0463 |
| INSURANCE_STATUS | 4 | 198.6011 | <.0001 |
| YEAR_OF_DIAGNOSIS | 1 | 1.6705 | 0.1962 |
| YEAR_OF_DIAGNOSIS-2009 | 1 | 0.6778 | 0.4103 |

Supplementary table 2.2 NSCLC Hispanic Patients

| Type 3 Analysis of Effects | | | |
| --- | --- | --- | --- |
| Effect | DF | Wald Chi-Square | Pr > ChiSq |
| FACILITY_TYPE_CD | 3 | 2025.4307 | <.0001 |
| FACILITY_LOCATION_CD | 8 | 425.3574 | <.0001 |
| SEX | 1 | 1250.6617 | <.0001 |
| age | 3 | 1576.7691 | <.0001 |
| CDCC_TOTAL_BEST | 2 | 3308.9906 | <.0001 |
| UR_CD_13 | 2 | 62.1278 | <.0001 |
| MED_INC_QUAR_12 | 3 | 23.4481 | <.0001 |
| NO_HSD_QUAR_12 | 3 | 24.4954 | <.0001 |
| INSURANCE_STATUS | 4 | 2289.4832 | <.0001 |
| YEAR_OF_DIAGNOSIS | 1 | 337.6794 | <.0001 |
| YEAR_OF_DIAGNOSIS-2009 | 1 | 237.2237 | <.0001 |

Supplementary table 2.3 NSCLC in Non-Hispanic White Patients

| Type 3 Analysis of Effects | | | |
| --- | --- | --- | --- |
| Effect | DF | Wald Chi-Square | Pr > ChiSq |
| FACILITY_TYPE_CD | 3 | 5.9787 | 0.1127 |
| FACILITY_LOCATION_CD | 8 | 40.8714 | <.0001 |
| age | 3 | 60.0965 | <.0001 |
| CDCC_TOTAL | 2 | 45.2606 | <.0001 |
| UR_CD_13 | 2 | 2.4076 | 0.3001 |
| MED_INC_QUAR_12 | 3 | 18.0762 | 0.0004 |
| NO_HSD_QUAR_12 | 3 | 9.3522 | 0.0250 |
| INSURANCE_STATUS | 4 | 1409.0099 | <.0001 |
| YEAR_OF_DIAGNOSIS | 1 | 236.2610 | <.0001 |
| YEAR_OF_DIAGNOSIS-2009 | 1 | 67.5473 | <.0001 |

Supplementary table 2.4 Breast African American Patients

| Type 3 Analysis of Effects | | | |
| --- | --- | --- | --- |
| Effect | DF | Wald Chi-Square | Pr > ChiSq |
| FACILITY_TYPE_CD | 3 | 40.6464 | <.0001 |
| FACILITY_LOCATION_CD | 8 | 29.9073 | 0.0002 |
| age | 3 | 27.7870 | <.0001 |
| CDCC_TOTAL | 2 | 11.9609 | 0.0025 |
| UR_CD_13 | 2 | 6.5597 | 0.0376 |
| MED_INC_QUAR_12 | 3 | 3.7251 | 0.2927 |
| NO_HSD_QUAR_12 | 3 | 2.0769 | 0.5566 |
| INSURANCE_STATUS | 4 | 318.3337 | <.0001 |
| YEAR_OF_DIAGNOSIS | 1 | 19.4791 | <.0001 |
| YEAR_OF_DIAGNOSIS-2009 | 1 | 2.0471 | 0.1525 |

Supplementary table 2.5 Breast Hispanic Patients

| Type 3 Analysis of Effects | | | |
| --- | --- | --- | --- |
| Effect | DF | Wald Chi-Square | Pr > ChiSq |
| FACILITY_TYPE_CD | 3 | 110.7511 | <.0001 |
| FACILITY_LOCATION_CD | 8 | 430.6742 | <.0001 |
| age | 3 | 296.2568 | <.0001 |
| CDCC_TOTAL | 2 | 646.5414 | <.0001 |
| UR_CD_13 | 2 | 35.7745 | <.0001 |
| MED_INC_QUAR_12 | 3 | 1.7025 | 0.6364 |
| NO_HSD_QUAR_12 | 3 | 310.1669 | <.0001 |
| INSURANCE_STATUS | 4 | 5499.5249 | <.0001 |
| YEAR_OF_DIAGNOSIS | 1 | 845.9282 | <.0001 |
| YEAR_OF_DIAGNOSIS-2009 | 1 | 313.1920 | <.0001 |

Supplementary table 2.6 Breast Non-Hispanic White patients

| Type 3 Analysis of Effects | | | |
| --- | --- | --- | --- |
| Effect | DF | Wald Chi-Square | Pr > ChiSq |
| FACILITY_TYPE_CD | 3 | 44.9043 | <.0001 |
| FACILITY_LOCATION_CD | 8 | 69.5644 | <.0001 |
| age | 3 | 1249.1319 | <.0001 |
| CDCC_TOTAL_BEST | 2 | 330.7473 | <.0001 |
| UR_CD_13 | 2 | 27.8655 | <.0001 |
| MED_INC_QUAR_12 | 3 | 65.3129 | <.0001 |
| NO_HSD_QUAR_12 | 3 | 11.9971 | 0.0074 |
| INSURANCE_STATUS | 4 | 2673.6830 | <.0001 |
| YEAR_OF_DIAGNOSIS | 1 | 7.9410 | 0.0048 |
| YEAR_OF_DIAGNOSIS-2009 | 1 | 105.9269 | <.0001 |

Supplementary table 2.7 Prostate African American Patients

| Type 3 Analysis of Effects | | | |
| --- | --- | --- | --- |
| Effect | DF | Wald Chi-Square | Pr > ChiSq |
| FACILITY_TYPE_CD | 3 | 20.0265 | 0.0002 |
| FACILITY_LOCATION_CD | 8 | 147.3562 | <.0001 |
| age | 3 | 293.0570 | <.0001 |
| CDCC_TOTAL_BEST | 2 | 143.5393 | <.0001 |
| UR_CD_13 | 2 | 1.4345 | 0.4881 |
| MED_INC_QUAR_12 | 3 | 2.7531 | 0.4313 |
| NO_HSD_QUAR_12 | 3 | 12.2834 | 0.0065 |
| INSURANCE_STATUS | 4 | 914.7542 | <.0001 |
| YEAR_OF_DIAGNOSIS | 1 | 1.0076 | 0.3155 |
| YEAR_OF_DIAGNOSIS-2009 | 1 | 19.5968 | <.0001 |

Supplementary table 2.8 Prostate Hispanic Patients

| Type 3 Analysis of Effects | | | |
| --- | --- | --- | --- |
| Effect | DF | Wald Chi-Square | Pr > ChiSq |
| FACILITY_TYPE_CD | 3 | 265.8510 | <.0001 |
| FACILITY_LOCATION_CD | 8 | 631.2918 | <.0001 |
| age | 3 | 5887.2812 | <.0001 |
| CDCC_TOTAL_BEST | 2 | 1817.5576 | <.0001 |
| UR_CD_13 | 2 | 106.4666 | <.0001 |
| MED_INC_QUAR_12 | 3 | 60.4440 | <.0001 |
| NO_HSD_QUAR_12 | 3 | 116.2356 | <.0001 |
| INSURANCE_STATUS | 4 | 4573.7468 | <.0001 |
| YEAR_OF_DIAGNOSIS | 1 | 23.6323 | <.0001 |
| YEAR_OF_DIAGNOSIS-2009 | 1 | 389.2309 | <.0001 |

Supplementary table 2.9 Prostate Non-Hispanic White

Supplementary Table 2. Analysis of effect was conducted for each ethnicity and cancer type to identify the significant predictor of metastatic cancer presentation.
